# Human-impacted landscapes facilitate hybridization between a native and an introduced tree

**DOI:** 10.1111/j.1752-4571.2012.00250.x

**Published:** 2012-11

**Authors:** Sean M Hoban, Tim S McCleary, Scott E Schlarbaum, Sandra L Anagnostakis, Jeanne Romero-Severson

**Affiliations:** 1 Department of Biological Sciences, University of Notre DameNotre Dame, IN, USA; 2 Department of Forestry, Wildlife and Fisheries, The University of TennesseeKnoxville, TN, USA; 3 The Connecticut Agricultural Experiment StationNew Haven, CT, USA

**Keywords:** anthropogenic disturbance, interspecific hybridization, introgression, *Juglans*

## Abstract

Spatial and temporal dynamics of hybridization, in particular the influence of local environmental conditions, are well studied for sympatric species but less is known for native-introduced systems, especially for long-lived species. We used microsatellite and chloroplast DNA markers to characterize the influence of anthropogenic landscapes on the extent, direction, and spatial distribution of hybridization between a native North American tree *Juglans cinerea* (butternut) and an introduced tree *Juglans ailantifolia* (Japanese walnut) for 1363 trees at 48 locations across the native range of butternut. Remarkably, admixture in anthropogenic sites reached nearly 70%, while fragmented and continuous forests showed minimal admixture (<8%). Furthermore, more hybrids in anthropogenic sites had *J. ailantifolia* seed parents (95%) than hybrids in fragmented and continuous forests (69% and 59%, respectively). Our results show a strong influence of landscape type on rate and direction of realized gene flow. While hybrids are common in anthropogenic landscapes, our results suggest that even small forested landscapes serve as substantial barriers to hybrid establishment, a key consideration for butternut conservation planning, a species already exhibiting severe decline, and for other North American forest trees that hybridize with introduced congeners.

## Introduction

Hybridization between introduced and native taxa may result in competitive exclusion of the native taxa and loss of native diversity (Levin et al. 1996; Rhymer and Simberloff 1996). Hybridization may also play a role in the origin of novel traits, weakening biological controls targeted to the introduced species or altering ecosystem function (Fritz 1999; Gaskin and Kazmer 2009). Alternatively, hybridization may introduce useful adaptive traits, such as resistance to exotic pests and diseases (Adams et al. 2002) or tolerance to new climatic conditions (Schweitzer et al. 2002). The extent to which non-native genes introgress into native populations depends on the frequency of hybridization, the fertility of hybrid offspring, and the relative fitness of hybrids and parental species across the locations in which hybridization occurs (Petit et al. 2004; Hails and Morley 2005). Hybridization studies often focus on the viability of early-generation hybrids under controlled conditions but in nature, studies on annual plants (Johansen-Morris and Latta 2008), fish (Gunnell et al. 2008), salamanders (Fitzpatrick and Shaffer 2007), and crustaceans (Brede et al. 2009) have shown that relative fitness of hybrids and parental species across different environments is the primary determinant of the ultimate extent of local introgression (Rubidge and Taylor 2004; Fitzpatrick and Shaffer 2007).

Many tree taxa introduced to North America are capable of hybridizing with native congeners, including taxa in *Pinus*, *Liquidambar*, *Morus*, *Populus*, *Juglans*, *Castanea*, and *Ulmus* (Wen 1999), genera whose roles in resource cycling and mast production may shift as hybridization alters genetic, demographic, and ecological processes. The interaction between rapidly changing ecological conditions and hybridization has implications for both evolutionary theory and forest management. A greater understanding of the role of local conditions on hybridization dynamics in long-lived taxa will enable better prediction of forest management outcomes and shed light on the impact of acute and prolonged disturbance on existing forest communities (Bleeker et al. 2007; Ellstrand 2009).

Butternut (*Juglans cinerea* L.), a North American forest tree, has experienced severe decline in the 20th century, primarily because of the fungal disease butternut canker (*Ophiognomonia clavigignenti-juglandacearum* (Nair, Kostichka & Kuntz) Broders & Boland (Oc-j) and habitat loss, and is currently under state and national protection (Fleguel 1996). Cultivars of Japanese walnut (*Juglans ailantifolia* Carrière) have been planted widely in orchards and farms in eastern North America since ∼1850. Naturally occurring F_1_ hybrids of Japanese walnut cultivars and butternut are such vigorous, fruitful trees (Ashworth 1969) that investigators have expressed concern over a possible range-wide genetic invasion (Ostry and Woeste 2004). As natural hybridization was first noted in the early 20th century (Ashworth 1969), contemporary regenerated forests, abandoned orchards, backyards, and suburban woodlots may contain naturalized Japanese walnuts and naturally occurring hybrid descendants.

As both species have relatively brief juvenile periods for trees (10–15 years), hybridization could have occurred over 6–10 generations. This historical situation provides us an opportunity to study interspecific hybridization and introgression across large spatial scales. In an initial survey of 187 trees, we detected natural hybridization in seven locations (Hoban et al. 2009). Admixture was extensive in two anthropogenic landscapes (92.5%) and limited (10.3%) in forested locations, suggesting that the ecosystem alterations that accompany agriculture and permanent settlements may influence the success of hybrids. However, the number of sites in this preliminary study was insufficient for statistical hypothesis testing.

Here, we have expanded our scope of investigation to the entire range (48 locations, *N* = 1415 individuals), utilized more DNA markers, and specifically addressed local conditions by examining trees in three landscapes: large (>1000 ha) continuous forest sites with minimal development, smaller forest (25–1000 ha) fragments, and anthropogenic sites (fencerows, pastures, and wooded patches <1 ha). We quantify the influence of landscape for three parameters: frequency of hybridization, direction of introgression, and spatial aggregation of hybrids where found. Frequency, directionality, and spatial extent of gene flow between the two species and their hybrids may influence how the native gene pool is retained within populations, and the geographic spread of introgression to new populations (Currat et al. 2008; Thompson et al. 2010). Each of these aspects of the hybridization process may be influenced by local conditions. Anthropogenic landscapes may serve as an introduction source (Fitzpatrick and Shaffer 2007), facilitate colonization (Culley and Hardiman 2009) by both parental species, or promote conditions that alter the relative fitness of parental species and hybrids (Martin et al. 2006), any of which could affect the extent and direction of gene exchange. Disturbed landscapes can also influence spatial distribution of hybrids through alteration in wind patterns (Hamzeh et al. 2007; Milne and Abbott 2008), recruitment sites, and the behavior and abundance of seed-dispersing animals (Burgess et al. 2005). We designed our study to test two hypotheses: (1) the incidence of hybrids and the direction of gene flow is unrelated to anthropogenic disturbance and (2) hybrids are randomly located (i.e., not spatially clustered) within the populations in which they occur.

## Materials and methods

### Species

*J. cinerea* (butternut) is a wind-pollinated, outcrossing North American tree, occurring primarily in riparian forest and human-impacted landscapes (orchards, woodlots, old fields). Individuals typically live <70 years, a relatively short time for forest trees (Fleguel 1996). Self-pollination is expected to be rare as butternut is heterodichogamous (male flowers and female flowers mature at different times on the same tree). The fruit encasing the seed is large (5–10 cm by 3–6 cm).

**Figure 1:**
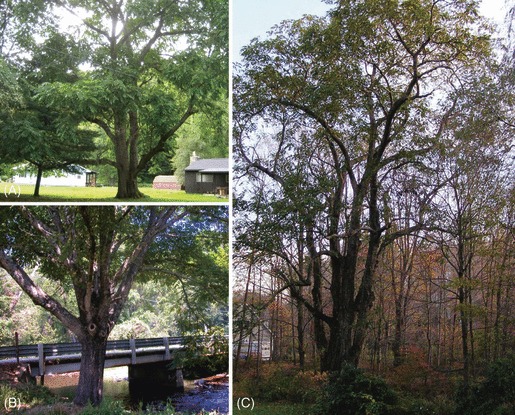
Trees in anthropogenic landscapes, to demonstrate the open nature of this habitat and its proximity to nearby forest. (A) F_1_ hybrid, hunting camp in Pennsylvania, (B) *Juglans ailantifolia*, roadside stream in western North Carolina, (C) F_1_ hybrid, woodlot in Connecticut.

Japanese walnut was introduced to North America as early as mid-19th century. By 1930, Japanese walnut had been planted in at least 30 states and eight provinces (Neilson 1930; Reed and Davidson 1954) and has since naturalized in woodlots, pastures (Fig. 1), and abandoned fields (Hoban et al. 2009). The reproductive biology of the two species is similar and phenologies overlap extensively (McDaniel 1956). Trees having the phenotypic characters of both species, as well as vigorous growth and remarkable reproductive output, have been reported many times (Gellatly 1966). Morphological characters (Ross-Davis et al. 2008) do not enable identification of all hybrids and are unreliable for generations beyond the F_1_.

While growers recognize the vigor of hybrids, there are few commercially viable hybrids in contrast to >50 established varieties of *J. ailantifolia*. Hybrids do not have the attractive heart-shaped nuts and easily cracked shells of the *J. ailantifolia* cultivars. Based on this and our visits to the orchards of hobbyists, retail growers, and nurseries in the United States and Canada, *J. ailantifolia* substantially outnumbers hybrids in cultivated settings.

The native range of *J. cinerea* overlaps with one other *Juglans* species, eastern black walnut (*J. nigra* L.). Despite intense study of both species, there is no confirmed instance of hybridization between these two species (Neilson 1930), suggesting strong or complete reproductive isolation. Black walnut is phenotypically distinct, and we did not sample any black walnuts. Persian walnut (*J. regia* L., native to central Asia) may hybridize with *J. cinerea* (Ostry and Woeste 2004), but *J. regia* is rarely planted within the native range of *J. cinerea*, because of butternut canker susceptibility, winterkill, and barrenness from early spring frosts. In our collections, we never observed naturalized *J. regia* individuals. *J. ailantifolia* is therefore likely to be the only species currently hybridizing with butternut, making our study simpler than those in which three or more species potentially interbreed (Thompson et al. 2010).

### Collections

**Figure 2:**
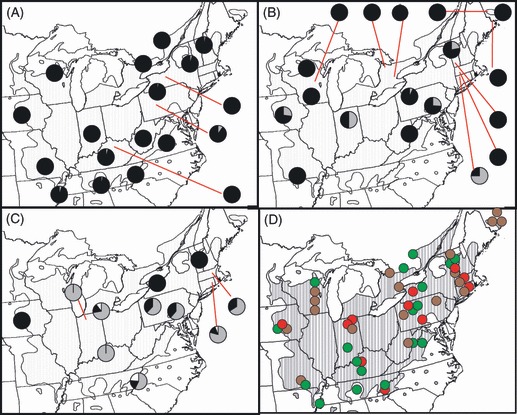
Pie charts for incidence of admixture, black = *Juglans cinerea*, white = *Juglans ailantifolia*, gray = hybrid. (A) continuous forest; (B) fragmented forest; (C) anthropogenic landscapes. (D) site locations, green = continuous forest, brown = fragmented forest, red = anthropogenic landscape, gray stripes = native range of *J. cinerea.*

We collected leaf or twig samples from the 24 germplasm repositories, arboreta, botanic gardens, or nurseries in the United States or Canada, which granted us permission to collect *J. cinerea* (*N* = 113), *J. ailantifolia* (*N* = 181), or hybrid (*N* = 16). The goal was to collect as many putative *J. ailantifolia* as possible, to help inform the Bayesian hybrid analysis, in which parental populations are not necessary but improve inference. We then collected samples from 1415 trees in 48 sites from across the native range (Table 1, Fig. 2). At each site, we collected most or all of trees that met the morphological criteria for butternut, heartnut, or hybrids. As we did not preferentially collect either hybrids or the parental species, the proportions of *J. ailantifolia* and hybrids we identified should be indicative of the actual incidence in nature. As the local frequency of *J. ailantifolia* has likely changed since the widespread local introductions facilitated by mail-order catalogs and extensive scion trading among growers, we cannot estimate the original number of *J. ailantifolia* introduced in any location. Most of the trees we sampled exceeded 5 cm diameter at breast height (DBH, 1.4 m from the ground), the size at which these species are typically reproductively capable. Approximately 5% of trees collected could be classified as juveniles (<5 cm DBH).

**Table 1:** Incidence of *Juglans cinerea*, hybrids, and *Juglans ailantifolia* by site

Landscape type	Site	S/P	*N*	H	A
Continuous forest	Scattered	PA	40	4	0.1
	Saint Francis	AR	39	2	0.05
	Barre/Berlin	VT	22	1	0.05
	Mammoth cave	KY	68	3	0.04
	Butternut valley	TN	168	5	0.03
	Green mountain	VT	30	1	0.03
	Allegheny	PA	37	1	0.03
	Ozarks	MO	129	0	0
	Chequamegon	WI	28	0	0
	Renfrew*	ON	26	0	0
	Peterborough*	ON	29	0	0
	G. Washington	WV	14	0	0
	Shenandoah	VA	32	0	0
	Cherokee	TN	1	0	0
	Bernheim	KY	1	0	0
	Finger lakes	NY	3	0	0
	Hoosier	IN	6	0	0
	Boone county	IA	1	0	0
	Total		674	17	0.025 (0.0)
Fragmented forest	State forests	CT	4	3	0.75
	Private forests	IN	4	2	0.5
	State parks	IA	11	3	0.27
	Various	PA	31	8	0.26
	Jericho	VT	27	6	0.26
	Allegheny	PA	88	6 (1)	0.068 (0.011)
	Putney*	VT	14	0	0.07
	Ozarks	MO	1	0	0
	Waupaca*	WI	20	0	0
	Whitewater*	WI	40	0	0
	Nottawasaga	ON	24	0	0
	Gilbert island	NB	39	0	0
	Keswick ridge	NB	33	0	0
	Blackville*	NB	41	0	0
	Franklin*	WV	22	0	0
	Holyoke range	MA	1	0	0
	Simcoe county	ON	7	0	0
	Hartman lake	WI	1	0	0
	Total		408	29 (1)	0.072 (0.002)
Anthropogenic	South bend	IN	1	1	1
	Bernheim	KY	3	3	1
	Rural, suburban	CT	82	65 (5)	0.793 (0.061)
	Rural, suburban	IN	29	21 (3)	0.724 (0.103)
	Rural, suburban	NC	21	12 (5)	0.571 (0.238)
	Rural, suburban	MA	50	33	0.66
	Scattered	PA	45	28	0.62
	Allegheny	PA	31	19	0.61
	Putney	VT	10	0	0
	Rural, suburban	IA	7	0	0
	Panama	NY	1	0	0
	Seattle*	WA	1	0	
	Total		281	182(13)	0.648 (0.046)

S/P, state/ province; *N*, number collected; H (JA), number of hybrids; number of *J. ailantifolia* in parentheses, A, proportion hybrids; proportion *J. ailantifolia* in parentheses.

* Nearest town, site on private property.

### Genotyping

Samples were genotyped at 12 highly polymorphic (see Results) nuclear microsatellite loci and at least two species-specific chloroplast markers, as previously described (Hoban et al. 2008; McCleary et al. 2009). The chloroplast markers are cleaved amplified polymorphic markers from the *psa*A-*trn*S and *trn*F-*trn*V regions containing species-specific single-nucleotide polymorphisms (SNPs). Amplification of a short (300–600 bp) sequence, followed by a restriction digest that cuts at the SNP, reveals one unrestricted fragment or several smaller restricted fragments visible on agarose gel (McCleary et al. 2009). Nuclear microsatellite markers were chosen from 34 candidate dinucleotide repeat containing sequences from butternut, selected for consistent amplifiability, ease of scoring, and polymorphism. We have previously tested these markers for null alleles, zygotic or Hardy–Weinberg equilibrium (HWE), and gametic (linkage) equilibrium in natural populations (Hoban et al. 2008, 2010). We did not test for HWE here because recent admixture is a violation of the assumptions for equilibrium, so HWE is not expected. Before analysis, we used Cervus (Kalinowski et al. 2007) to identify and remove the duplicate genotypes that arise in natural populations from stump sprouting and in cultivation from grafting. Duplicates bias the allele frequencies used to define a species in admixture analysis, similar to the influence of family groups (Anderson and Dunham 2008).

### Hybrid analysis

We used the Bayesian approach in NewHybrids (Anderson and Thompson 2002) to infer allele frequencies for each species and assign individuals to one of six classes: *J. cinerea*, *J. ailantifolia*, F_1_ hybrid, F_2_ hybrid, F_1_ backcross to *J. cinerea* (BC_JC_), and F_1_ backcross to *J. ailantifolia* (BC_JA_), with a probability cutoff of 0.75 in a given category for making an assignment. We explored the effects of using cutoffs of 0.90 and 0.95 with a simulation study, explained below. Individuals not assigned to any single category at *P* > 0.75 were assigned to a ‘mix’ hybrid category. The ‘mix’ category represents individuals whose recovery of parental alleles is outside the expected range for the four hybrid classes, the most likely explanation being that the individual is a complex hybrid (e.g., backcross × F_1_). We expected to find a number of such individuals, given the time since first introduction. *A priori*, we cannot predict the relative proportion of early- and later-generation hybrids. This depends on hybrid fertility and abundance. If hybrids are typically reproductively unsuccessful, we expect most naturally occurring hybrids to be the F_1_ generation, but if hybrids produce fertile offspring, later-generation hybrids are expected. The high numbers of viable nuts observed in hybrids makes the later prediction more plausible, but quantification of hybrid fitness logically follows after we ascertain whether and where hybrids exist.

For program settings, we used Jeffrey’s prior on pi and theta (recommended for microsatellites), 100 000 steps for the burn-in and 500 000 steps for the MCMC. The z option (to identify known individuals) was not used. We also performed analysis using the uniform prior for comparison. Several investigations (Streiff et al. 2005; Vähä and Primmer 2006; Wallace 2006), including our previous work (Hoban et al. 2009), have used both NewHybrids and the Bayesian clustering program Structure (Pritchard et al. 2000) to identify hybrids and have found highly concordant results. We use NewHybrids alone in this investigation because of its higher accuracy (Burgarella et al. 2009) and ability to identify F_1_, F_2_, and backcross individuals with a given probability.

### Simulation study

To test the validity of NewHybrids assignments, at several thresholds, we performed a simulation study using hybridlab (Nielsen et al. 2006) to create five replicated sets of genotype data with 100 *in silico* individuals in each category: F_1_, F_2_, BC_JC_, and BC_JA_, comparable to our dataset. For parental allele frequencies, we used 994 individuals from our observed dataset that had a posterior probability >0.9985 of being *J. cinerea* and 66 individuals that had a posterior probability >0.99 of being *J. ailantifolia*. We used NewHybrids to analyze each of the five simulated datasets and calculated efficiency and accuracy (Vähä and Primmer 2006), for cutoff values of 0.75, 0.90, and 0.95.

### Landscape assignment

Each collection location was assigned a landscape: continuous forest, fragmented forest, or anthropogenic (Table 1). While landscapes exist on a continuum between these categories, assignments were based as follows. Continuous forest sites (typically National Park or National Forest sites) were characterized by large (>1000 ha) tracts of forest with minimal development outside of access roads and hiking trails. Fragmented forest sites were smaller (25–1000 ha) and typically occurred as protected woodlots and nature preserves in an agricultural matrix. Anthropogenic sites included yards, small parks, fencerows, pastures, and roadsides. Thompson et al. (2010) recently used similar broad categories. The forested landscape was represented by 18 locations (*N* = 674), the fragmented landscape by 18 locations (*N* = 408), and the anthropogenic landscape by 12 locations (*N* = 281, Table 1). As most forests in eastern North America were logged, burned, and farmed prior to 1900 (Williams 1989), our designation reflects the condition of the last 50–100 years.

### Comparison of landscape types

To test the null hypothesis that landscapes have equal rates of hybridization, we used a Fisher’s exact test to compare landscape types for counts of non-*J. cinerea* individuals. To test the hypothesis that landscape types have the same proportions of each hybrid category (e.g., F_1_, F_2_), we used a Fisher’s exact test to compare counts in each category, in each type. To quantify variation among sites within categories relative to between categories, we performed an anova with landscape as an independent variable, and admixture as the response variable. We also performed an anova on proportion of hybrids with *J. ailantifolia* chloroplast as the response variable. The latter test was only performed for sites in which more than one hybrid was found (*N* = 17).

### Chloroplast identity in hybrids

To test the null hypothesis that the two species are equally likely as seed parents, we used Fisher’s exact tests to compare the number of hybrids with the *J. cinerea* chloroplast to the number with the *J. ailantifolia* chloroplast. To test the hypothesis that landscape type has no influence, we performed the same test to compare counts across landscapes and across hybrid classes. All tests were performed in R, Development Core Team (2005).

### Spatial clustering of hybrids

Within each continuous or fragmented forested population in which we found more than one hybrid (*N* = 6), we measured the pairwise geographic distance between all non-*J. cinerea* individuals (*D*_not-jc_) and between all *J. cinerea* individuals (*D*_jc_). For the four populations in which more than two hybrids were found, we compared *D*_not-jc_ to *D*_jc_ using *t*-tests. For the two populations in which only two hybrids were found, we used a *z*-test to compare *D*_not-jc_ with *D*_jc_.

## Results

We genotyped 1725 trees and identified 210 duplicate genotypes (102 *J. ailantifolia* reference, nine hybrid reference, 47 *J. cinerea* reference, and 52 naturally occurring trees). These were removed, leaving 1515 unique genotypes for analysis: 79 *J. ailantifolia* reference, seven hybrid reference, 66 *J. cinerea* reference, and 1363 naturally occurring trees.

We identified 356 alleles (maximum per locus = 69, minimum = 18, mean = 29.7). Mean observed heterozygosity across loci was 0.748. Marker loci showed strong allele frequency differences between species, and three were nearly diagnostic (WGA_82, B121, and B264, Fig. S1).

Among the 1363 naturally occurring trees, we identified 1121 *J. cinerea* (JC), 14 *J. ailantifolia* (JA), 96 F_1_ hybrids, 31 F_2_ hybrids, 42 BC_JC_, 11 BC_JA_, and 48 ‘mix’ (see Materials and methods for definition) hybrids. The mean probability with which individuals were assigned to a category was 0.997 (JC), 0.979 (JA), 0.961 (F_1_), 0.956 (F_2_), 0.892 (BC_JC_), and 0.855 (BC_JA_) (Fig. S2). Considering only those sites in which more than four trees were collected, the highest admixture observed was 10% in the continuous forested sites, 27.3% in the fragmented forested sites, and 79.3% in the anthropogenic landscapes. Results using the uniform prior were similar (Table S1). Our simulation study showed that the best overall performance for all categories is achieved with a 0.75 cutoff (Table S2). With this cutoff, accuracy is >0.95 for all categories except BC_JA_, for which it is 0.92. Efficiency is >0.97 for parental species and F_1_, 0.90 for BC_JC_, 0.75 for F_2_, and 0.89 for BC_JA_.

Landscapes significantly and dramatically differed for admixture (exact test *P* < 0.0001, Table 2). Excluding sites represented by fewer than ten trees, all anthropogenic sites showed >57% admixture, while all forested sites (continuous and fragmented) showed <28% admixture. Landscape did not influence the proportion of different classes of hybrids (exact test *P* = 0.205, Table 2). Overall, a striking majority of hybrids had the *J. ailantifolia* chloroplast (exact test *P* < 0.0001, Tables 3 and 4). Additionally, landscape types and hybrid classes showed significant differences for chloroplast type within hybrids (exact test *P* = 0.029) and for chloroplast type within hybrids across landscape types (exact test *P* < 0.0001, Table 3). Further, while variation is substantial within landscapes and hybrid categories, landscape type is a significant predictor variable for incidence of admixture and the incidence of hybrids having the JA chloroplast type (Table 5).

**Table 2:** Number of *Juglans cinerea* (JC), *Juglans ailantifolia* (JA), and hybrids by hybrid category and landscape type

Landscape type	JC	JA	F_1_	F_2_	BC_JC_	BC_JA_	Mix	I
Forest	657	0	1	3	7	0	6	0.025
Fragmented	378	1	14	6	4	1	4	0.072
Anthropogenic	86	13	81	22	31	10	38	0.648

BC_JC_, F_1_ backcross to *J. cinerea*; BC_JA_, F_1_ backcross to *J. ailantifolia*; I, incidence of hybrids.

**Table 3:** Incidence of hybrids having the *Juglans ailantifolia* chloroplast, by sites with hybrids

Landscape type	Site	S/P	*N*	H	H(JAcp)	I(JAcp)
Continuous forest	Scattered	PA	40	4	4	1.000
	Saint Francis	AR	39	2	1	0.500
	Barre/Berlin	VT	22	1	0	0.000
	Mammoth cave	KY	68	3	1	0.333
	Butternut valley	TN	168	5	2	0.400
	Green mountain	VT	30	1	1	1.000
	Allegheny	PA	37	1	1	1.000
Fragmented forest	State forests	CT	4	3	3	1.000
	Private forests	IN	4	2	1	0.500
	State parks	IA	11	3	3	1.000
	Various	PA	31	8	4	0.500
	Jericho	VT	27	6	4	0.800
	Allegheny	PA	88	6	5	0.833
Anthropogenic	South Bend	IN	1	1	1	1.000
	Bernheim	KY	3	3	2	0.667
	Rural, suburban	CT	82	65	61	0.953
	Rural, suburban	IN	29	21	20	0.952
	Rural, suburban	NC	21	12	12	1.000
	Rural, suburban	MA	50	33	29	0.879
	Scattered	PA	45	28	28	1.000
	Allegheny	PA	31	19	19	1.000

S/P, state/province; *N*, number of trees collected; H, number of hybrids; H (JAcp), number of hybrids with the *J. ailantifolia* chloroplast; I (JAcp), incidence of hybrids with the *J. ailantifolia* chloroplast.

**Table 4:** *Juglans cinerea* and *Juglans ailantifolia* chloroplast types for each hybrid class by landscape

Landscape type	cp*	F_1_	F_2_	BC_JC_	BC_JA_	Mix	T†	JCcp‡
Continuous forest	JC		2	1	0	4	7	0.412
	JA	1	1	6	0	2	10	
Fragmented	JC	1	4	1	0	3	9	0.310
	JA	13	2	3	1	1	20	
Anthropogenic§	JC	2	3	1	0	3	9	0.050
	JA	79	18	30	10	35	172	
Total¶	JC	3	9	3	0	10	25	0.110
	JA	93	21	39	11	38	202	

* Chloroplast type.

† Total by landscape and chloroplast type.

‡ Incidence of hybrids having the JC chloroplast by landscape type.

§ Chloroplast data missing for one F_2_ individual in the anthropogenic landscape.

¶ Sum across landscapes by genotypic class and chloroplast type.

**Table 5:** anovas for the effect of landscape type

Response variable	Source of variation	SS	df	MS	F	*P*-value
Incidence of admixture	Between landscapes	1.986	2	0.993	19.842	<0.001
	Within landscapes	2.202	44	0.050		
	Total	4.188	46			
Incidence of hybrids with JA chloroplast	Between landscapes	0.337	2	0.168	3.818	0.047
	Within landscapes	0.618	14	0.044		
	Total	0.955	16			

Spatial analysis of the six natural populations in which more than one hybrid was found (Table S3) revealed that in two populations, hybrids were clustered (distance between non-*J. cinerea* was significantly smaller than distance between *J. cinerea*). Both populations occurred in fragmented landscapes (Table S3, Fig. S3).

## Discussion

Hybridization between native and introduced congeners may occur rapidly and in all exposed populations (Metcalf et al. 2008), but rates usually vary in space (Gunnell et al. 2008) and time (Brede et al. 2009). In the first geographically extensive study of hybrid dynamics between a native and introduced forest tree in North America, we detected bidirectional and advanced-generation hybridization over a large geographic area. As advanced-generation hybrids are not available as nursery stock, these trees demonstrate that hybrids can produce descendants fit enough to mature and produce descendants of their own. Further, we show that hybridization occurs not as a regional hybrid front, but rather as pockets within anthropogenic landscapes across the range (Fig. 2). Lastly, we observed biased gene flow (most hybrids had a *J. ailantifolia* seed parent), with the bias occurring at significantly higher levels in anthropogenic landscapes. We conclude that while reproductive barriers between the species are porous, landscapes are clearly associated with the direction and extent of realized gene flow.

### High incidence of hybrids in anthropogenic sites: dispersal and introduction history

As late as 1984, no grafted F_1_ hybrid cultivars were available in the northeastern United States (Goodell 1984), suggesting that the high incidence of hybrids in the northeast, including advanced-generation hybrids, is a natural occurrence. We suspect that sites containing hybrids but not *J. ailantifolia* are the result of the natural death of the *J. ailantifolia* parents, similar to other findings (Lepais et al. 2009). *J. ailantifolia* and *J. cinerea* live 60–70 years under natural conditions and bear nuts from age 10–15 years until death. F_1_ hybrids could easily outlive their parents.

Our results suggest that the high incidence of hybrid trees in anthropogenic landscapes is attributed to a combination of introduction history, dispersal limitation, and reduced competition. Both species occur in anthropogenic landscapes, especially along fencerows, streams, and roadsides (Ostry and Pijut 2000; Hoover 1919; McDaniel 1956), open sites that may facilitate local hybrid recruitment. As seed dispersal is limited (Tamura and Hayashi 2008) and the requirement for light essential, successful hybrid establishment out of anthropogenic landscapes into neighboring forest, where competition for light and space is high, is likely rare. Consistent with this scenario, we observed a lower incidence of hybrids, and a higher representation of *J. ailantifolia* pollen parents in hybrids in forested landscapes. Our results in butternut, a heterodichogamous species with limited seed dispersal, and those of Thompson et al. (2010) on two native and one introduced *Populus* (a dioecious species with widespread seed dispersal) both show that hybrid individuals occur more frequently at sites with high anthropogenic disturbance. In contrast, hybrids between a native and an introduced elm (*Ulmus*) occurred at high frequency across a variety of landscapes (Zalapa et al. 2009) and hybrids between native and introduced *Morus* species occurred at high frequency in four forested landscapes (Burgess et al. 2005). Demography may explain the high rates of hybridization in the *Morus* studies, as the introduced species outnumbers the native species at the northern range margin of the native species, where the studies took place. Clearly, variation in hybridization rates is influenced by landscape context, reproductive biology, and propagule dispersal.

Investigators have considered the role of landscape in hybrid establishment and persistence for many years (Anderson 1948). However, many investigations attribute hybrid establishment and persistence to direct selection for stress tolerance such as escape from herbivory (Gaskin and Kazmer 2009), flood tolerance (Martin et al. 2006), or drought tolerance, (Rieseberg et al. 2003). In contrast, we demonstrate a primary role for introduction history, abundance, and dispersal limitation, as previously suggested (Gunnell et al. 2008) and demonstrated in *Populus* (Thompson et al. 2010). Seed dispersal and recruitment dynamics also play a role in *Eucalyptus* hybridization (Field et al., 2011).

Our results are also consistent with observations in both plant and animal taxa that hybridization is often asymmetric (Hamzeh et al. 2007; Metcalf et al. 2008; Milne and Abbott 2008). Thompson et al. (2010) and Burgess et al. (2005) observed a bias in backcrossing toward native species, consistent with our finding that most (∼80%) backcrosses were to *J. cinerea*. Consistent directional gene flow can lead to pollen swamping (Petit et al. 2004), and capture of organelle genomes, a possibility in ours and other systems (Floate 2004). Although cytonuclear or other reproductive incompatibilities may cause asymmetrical introgression (Landry et al. 2007; Conesa et al. 2008), our results are most consistent with a simple demographic model in which the more numerous (native in this case) species is the most likely pollinator (Burgess et al. 2005; Currat et al. 2008), as observed among hybridizing oaks (Lepais et al. 2009). Overall, we suggest that the establishment and persistence of hybrids in many plant taxa is determined more by introduction history, landscape features, and environmental differences than the degree of intrinsic incompatibility between species.

Our results may be partly because of other mechanisms. It is reasonable to hypothesize that Japanese walnut and hybrids are less adapted than butternuts to local forest conditions. Hybrid establishment may simply result from reduced competition for light and water in the open anthropogenic landscapes (Lexer et al. 2005). However, this does not explain the association of landscape with direction of hybridization. The most parsimonious mechanism, and most consistent with our data, is introduction history and the success of seed dispersal.

We speculate that the environmental variance of the continental climates in the Northern Hemisphere results in high genetic diversity within long-lived species with high reproductive outputs and high phenotypic plasticity within these individuals. Thus, forest trees from China, Europe, and North America may persist in any of these locations long enough to produce millions of pollen grains and many thousands of seeds, providing many opportunities to find the right combination of alleles that will result in fertile hybrids. The relative roles of landscape and intrinsic fitness in establishment and persistence of hybrids in forest trees merit additional investigation.

### Applied conservation implications

Butternut outnumbers hybrids in all forested locations, despite the time since introduction of *J. ailantifolia*, the vigor and prolificacy of hybrids, and disease pressure. Future spatial expansion of hybrids out of anthropogenic landscapes will likely proceed slowly, and even moderate loss of native genetic material to hybridization is unlikely. However, thresholds may exist after which hybridization rapidly expands or disappears (Hails and Morley 2005). Unfortunately, limited data on this process in forest trees make delimitation of this threshold difficult and firm statements regarding hybrid persistence require more comparative studies.

We identified no hybrids in Wisconsin or Canada. This could be due to limited introduction, a lower frequency of recent anthropogenic disturbance, or a low probability of *J. ailantifolia* and hybrid seed survival in colder climates. Analysis of additional Wisconsin and Canadian samples will reveal whether hybrids actually do occur much less frequently at the northern edges of the range for *J. cinerea*. A finer-scale examination of landscape, such as forest type (riparian/upland) or distance from commercial orchards, as in Sampson and Byrne (2008), may further clarify the circumstances under which establishment of hybrids is most likely.

### Evolutionary consequences

Most hybrids show high tolerance to the butternut canker disease (Ostry and Woeste 2004). However, some *J. cinerea* individuals have persisted even under heavy disease pressure. The disease progresses more slowly in these trees, suggesting a moderate level of tolerance. Many F_1_ hybrids may have a general lack of adaptation that is only slightly offset by the advantage of disease tolerance. As our study was based on successful, that is, mature trees, we did not capture the number of F_1_ seeds that failed to germinate or died before maturity. If additional studies indicate that more hybrid seedlings and juveniles die before reaching maturity than butternuts, this would contrast with results in *Morus* in which the native species was always least fit (Burgess and Husband 2006).

The vigor and size typical in early-generation hybrids may be lost in later generations along with disease tolerance. A necessary future direction is to quantify relative fitness under a range of disease and environmental conditions. This will also enable a balanced assessment of the potential for genetic improvement via hybridization. Given the relatively short generation time of these two species, ours could serve as model system for investigating the evolutionary dynamics of two hybridizing species and a pathogen, an increasingly common circumstance in North American forests (Cullingham et al. 2011).

We observed few *J. ailantifolia* individuals in any site (overall ∼1%), and in many locations where hybrids were identified, *J. ailantifolia* was not found. From this, we infer hybrids may persist long enough to outlive their parents. However, a large proportion of hybrids identified were first generation. If hybrids suffer a fitness disadvantage, they may be a demographic sink (Wolf et al. 2001), reducing overall fitness of the population. On the other hand, adaptive evolution in the hybrid population may be rapid if admixture-derived novel phenotypes lead to more successful, invasive hybrids (Campbell et al. 2006; Gaskin and Kazmer 2009). The high genetic diversity in both species could facilitate a rapid response to selection pressure. Mathematical models of population and disease dynamics, parameterized with observed census, admixture, and landscape characteristics, could explore long-term demographic and evolutionary outcomes in this and other systems, including cases where introduced species outnumber native congeners (Zheng et al. 2004; Sampson and Byrne 2008).

## Conclusions

Our results show that landscape is a key consideration in native–introduced hybrid population dynamics. While it is likely that a combination of introduction history, propagule dispersal dynamics, landscape suitability, and relative fitness ultimately determines the degree to which native alleles are retained in hybrid populations, we proffer that the first two play a major role and should be considered as a null hypothesis prior to invoking selection. We emphasize that the influence of seed dispersal opportunities and resources for seedling establishment may supersede the influence of selection by preventing hybrid establishment in the first place. If hybridization is frequently dispersal limited, as we suggest, the preservation and restoration of contiguous blocks of natural landscapes may form a partial barrier against genetic invasion, another ecological and evolutionary argument for *in situ* preservation of natural areas. Our results also suggest that more work is needed to compare hybridization dynamics in annual or biennial species with perennials. Lastly, this and other work indicate that in natural settings, Asian and European forest trees and other perennials remain capable of introgression via fertile F_1_ hybrids into North American congeneric taxa despite millions of years of separation. While presenting a great conservation challenge, this also presents opportunities for the study of speciation and ecological consequences of invasions.

## Data Archiving Statement

Data deposited in the Dryad repository: doi:10.5061/dryad.89735cr0.

## Supporting information

**Figure S1.** Allele frequencies by marker and species.

**Figure S2.** Histograms of probabilities for the NewHybrids assignments. *X*-axis is the lower bound of each probability bin.

**Figure S3.** Maps of forested sites showing clustering of hybrids.

**Table S1.** Counts in each category under the uniform and Jeffrey’s prior.

**Table S2.** Efficiency, accuracy and performance of NewHybrids on simulated datasets with different thresholds.

**Table S3.** Distances in clusters of hybrids.
