# Supplementary material for: Human-impacted landscapes facilitate hybridization between a native and an introduced tree
Source: Evol Appl. 2012 Nov;5(7):720–31. doi: 10.1111/j.1752-4571.2012.00250.x (PMC3492897; doi:10.1111/j.1752-4571.2012.00250.x)
Supplement: Supplementary file 1 [file eva0005-0720-SD1.pdf]

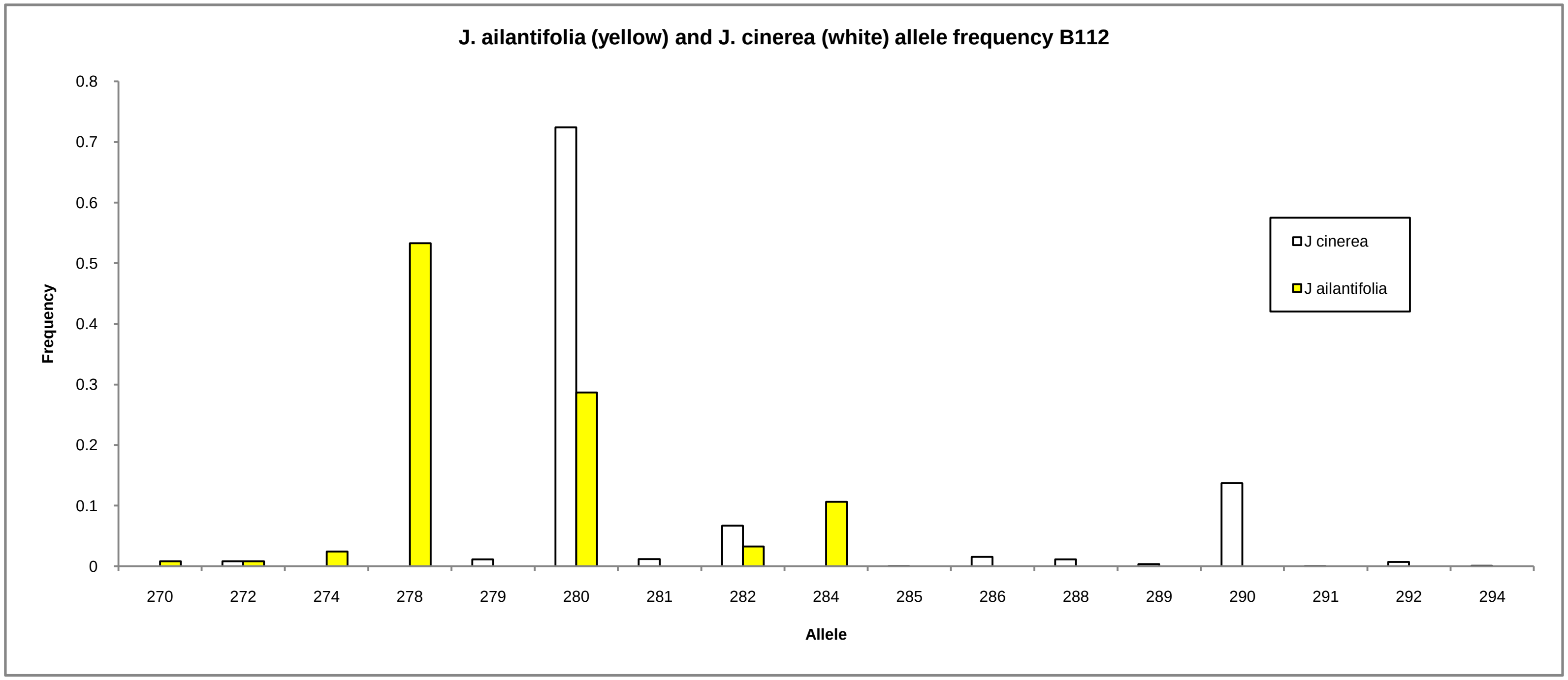

J. ailantifolia (yellow) and J. cinerea (white) allele frequency B112
Frequency
0.8
0.7
0.6
0.5
0.4
0.3
0.2
0.1
0
270 272 274 278 279 280 281 282 284 285 286 288 289 290 291 292 294
Allele
J cinerea
J ailantifolia

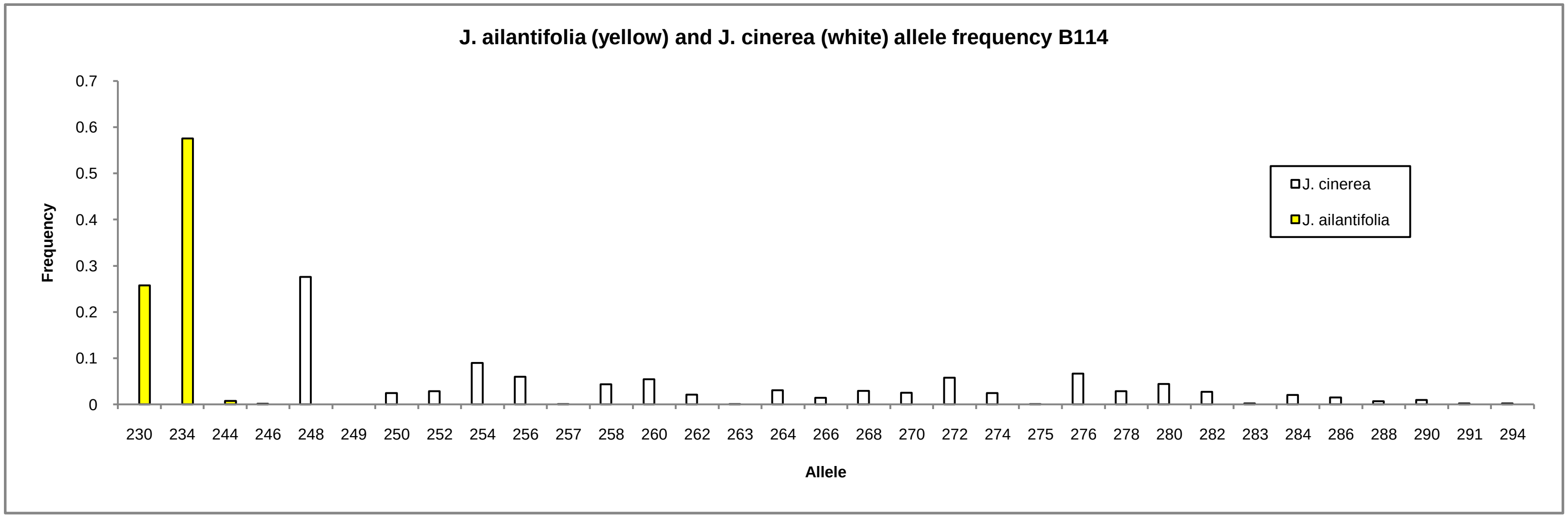

J. ailantifolia (yellow) and J. cinerea (white) allele frequency B114
Frequency
0.7
0.6
0.5
0.4
0.3
0.2
0.1
0
230 234 244 246 248 249 250 252 254 256 257 258 260 262 263 264 266 268 270 272 274 275 276 278 280 282 283 284 286 288 290 291 294
Allele
J. cinerea
J. ailantifolia

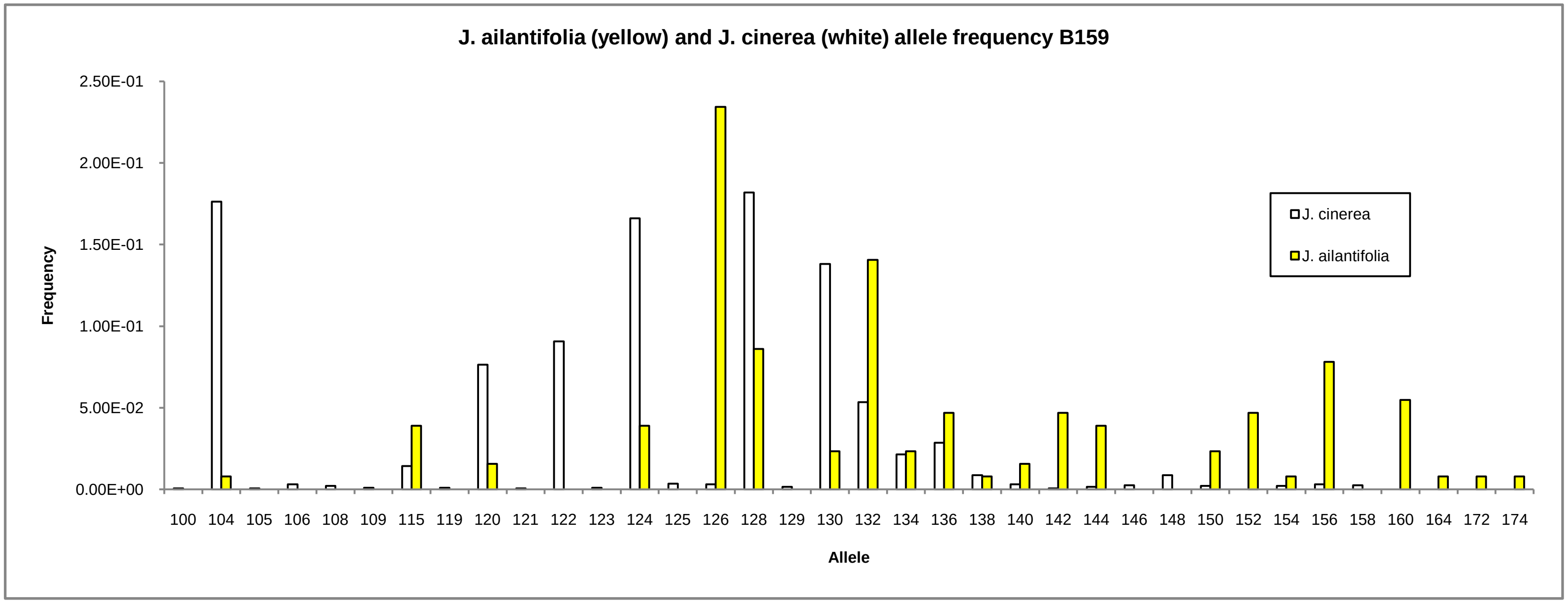
J. ailantifolia (yellow) and J. cinerea (white) allele frequency B159
Frequency
2.50E-01
2.00E-01
1.50E-01
1.00E-01
5.00E-02
0.00E+00
100 104 105 106 108 109 115 119 120 121 122 123 124 125 126 128 129 130 132 134 136 138 140 142 144 146 148 150 152 154 156 158 160 164 172 174
Allele
J. cinerea
J. ailantifolia

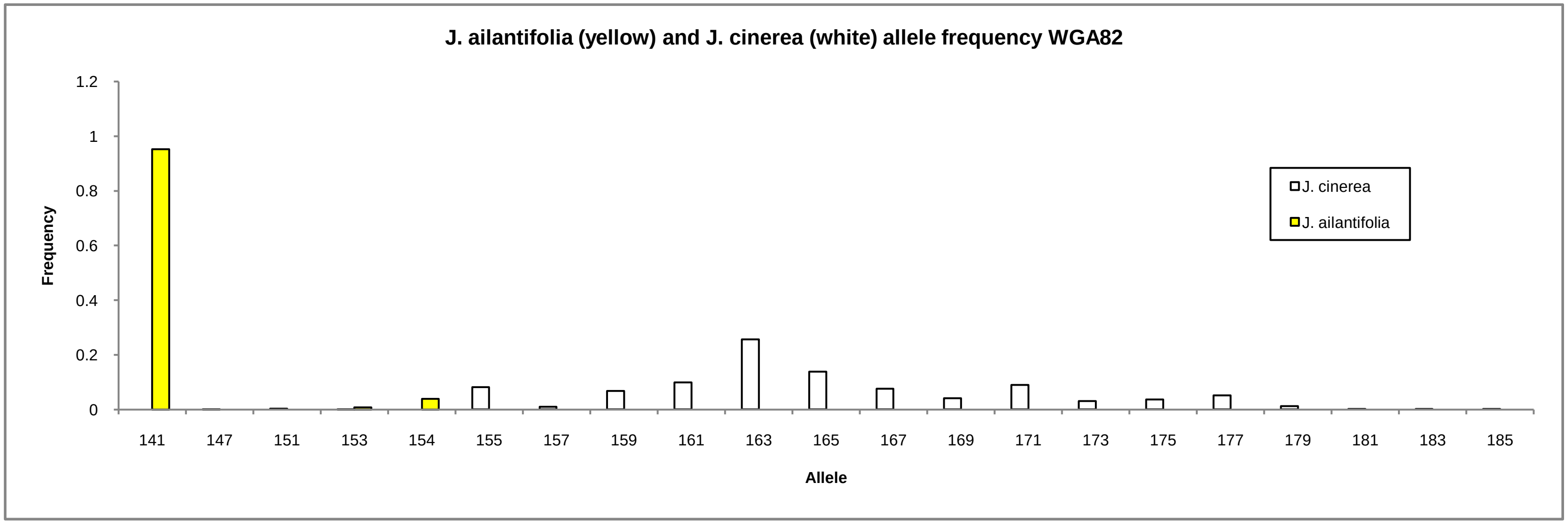
J. ailantifolia (yellow) and J. cinerea (white) allele frequency WGA82
Frequency
1.2
1
0.8
0.6
0.4
0.2
0
141 147 151 153 154 155 157 159 161 163 165 167 169 171 173 175 177 179 181 183 185
Allele
J. cinerea
J. ailantifolia

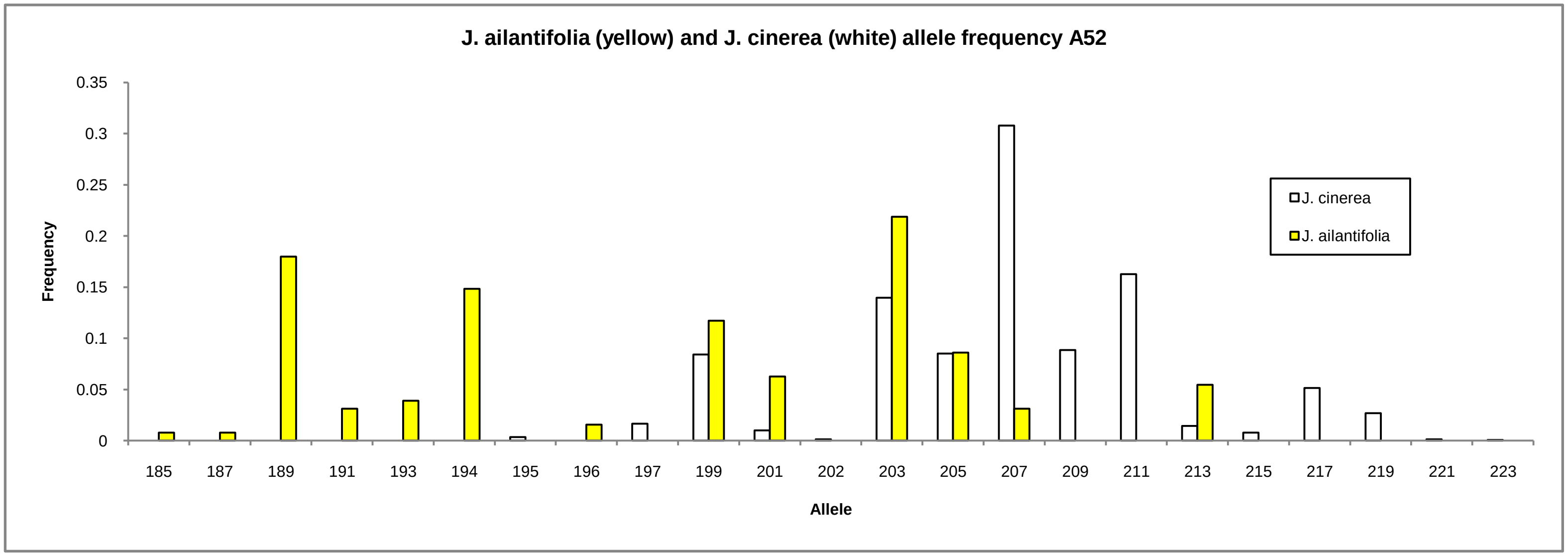
J. ailantifolia (yellow) and J. cinerea (white) allele frequency A52
Frequency
0.35
0.3
0.25
0.2
0.15
0.1
0.05
0
185 187 189 191 193 194 195 196 197 199 201 202 203 205 207 209 211 213 215 217 219 221 223
Allele
J. cinerea
J. ailantifolia

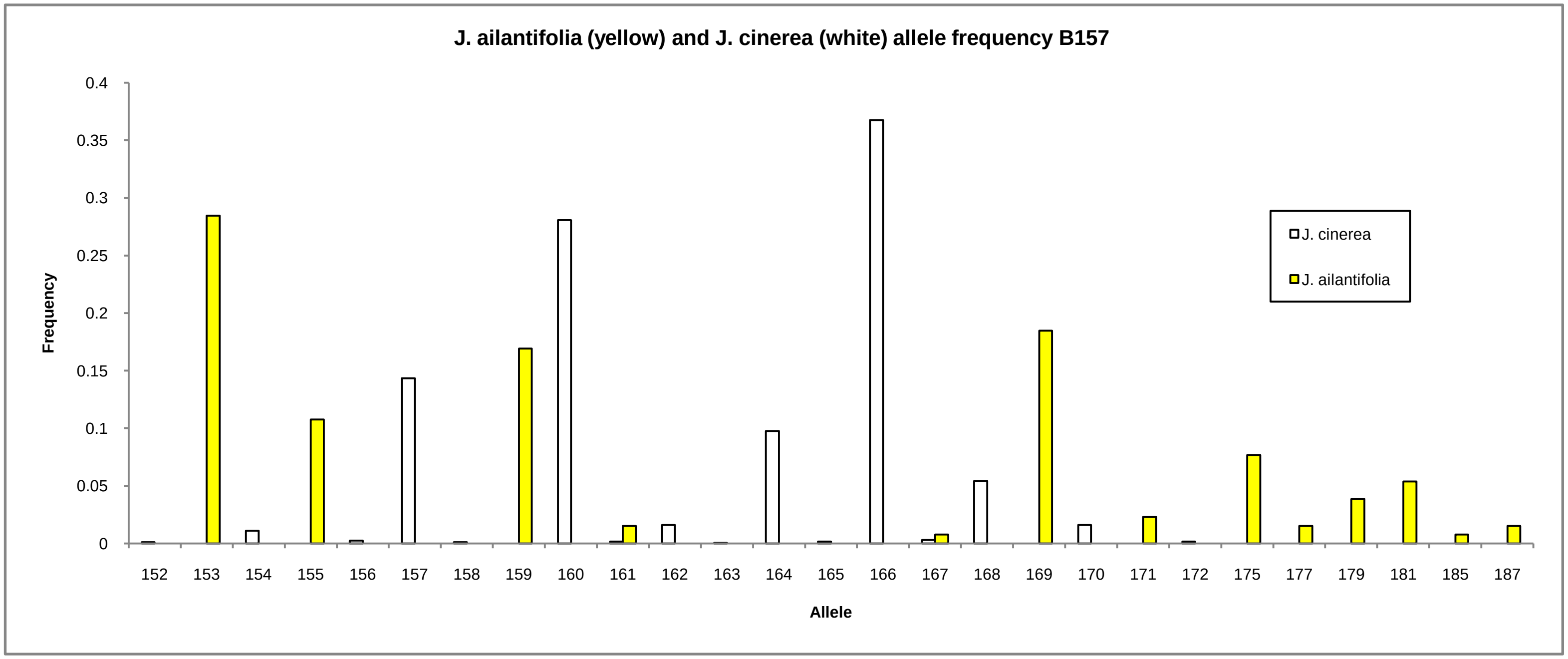
J. ailantifolia (yellow) and J. cinerea (white) allele frequency B157
Frequency
0.4
0.35
0.3
0.25
0.2
0.15
0.1
0.05
0
152 153 154 155 156 157 158 159 160 161 162 163 164 165 166 167 168 169 170 171 172 175 177 179 181 185 187
Allele
J. cinerea
J. ailantifolia

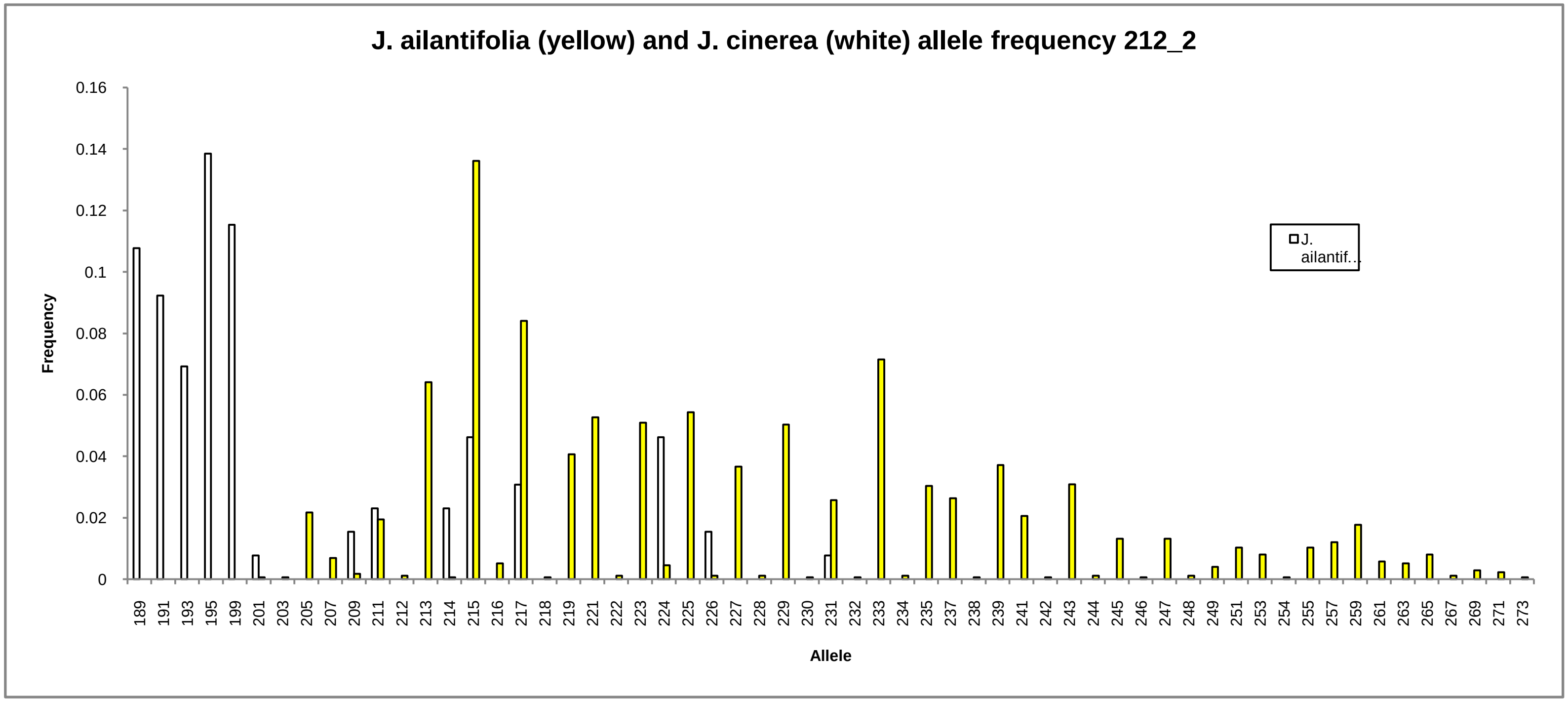
J. ailantifolia (yellow) and J. cinerea (white) allele frequency 212_2
Frequency
Allele
J. ailantif...

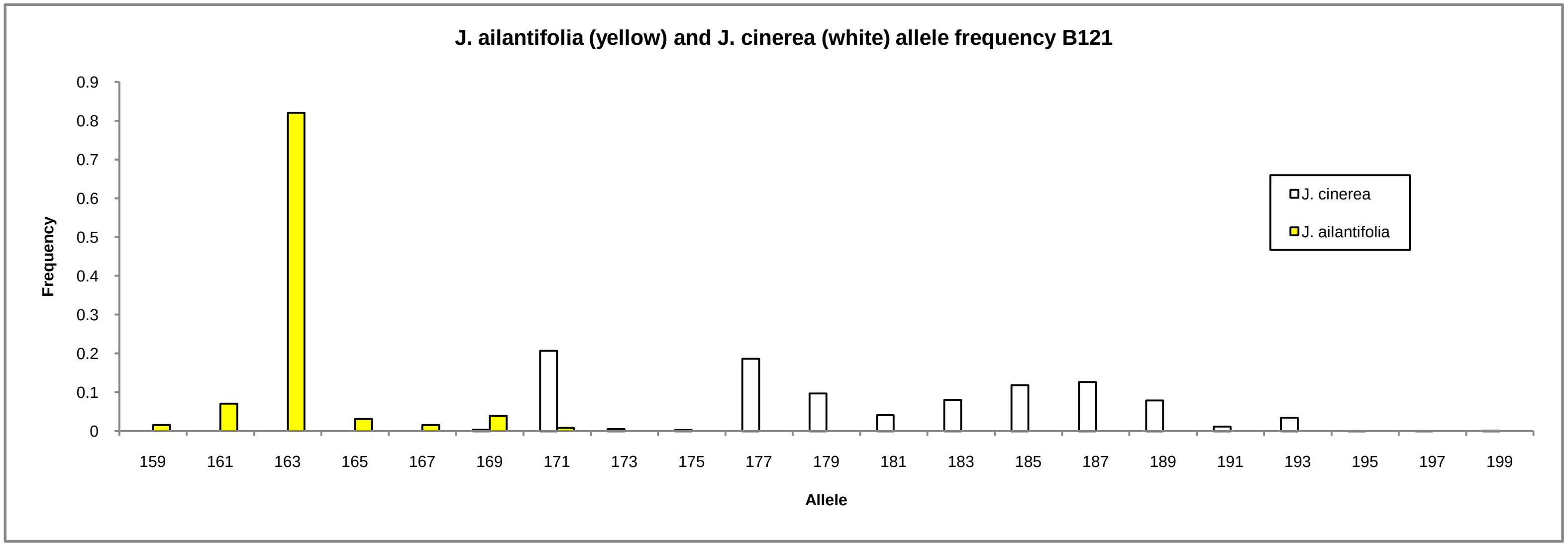
J. ailantifolia (yellow) and J. cinerea (white) allele frequency B121
Frequency
Allele
J. cinerea
J. ailantifolia

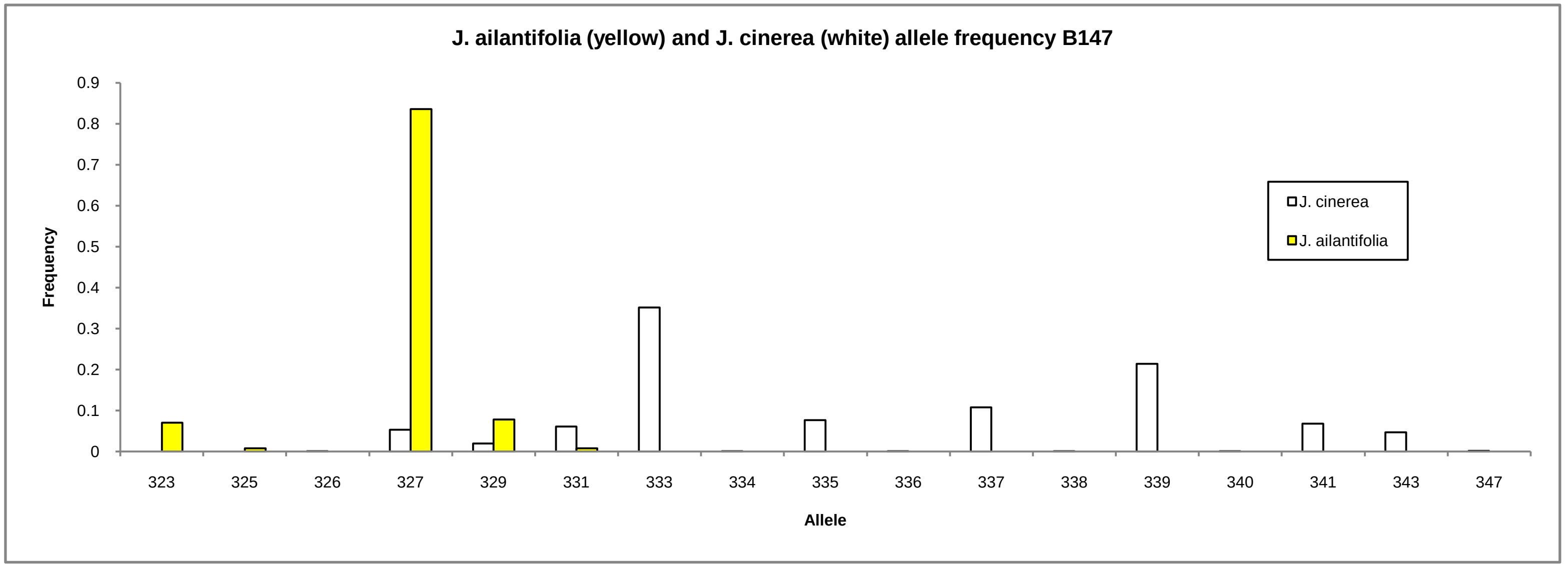
J. ailantifolia (yellow) and J. cinerea (white) allele frequency B147
Frequency
0.9
0.8
0.7
0.6
0.5
0.4
0.3
0.2
0.1
0
323
325
326
327
329
331
333
334
335
336
337
338
339
340
341
343
347
Allele
J. cinerea
J. ailantifolia

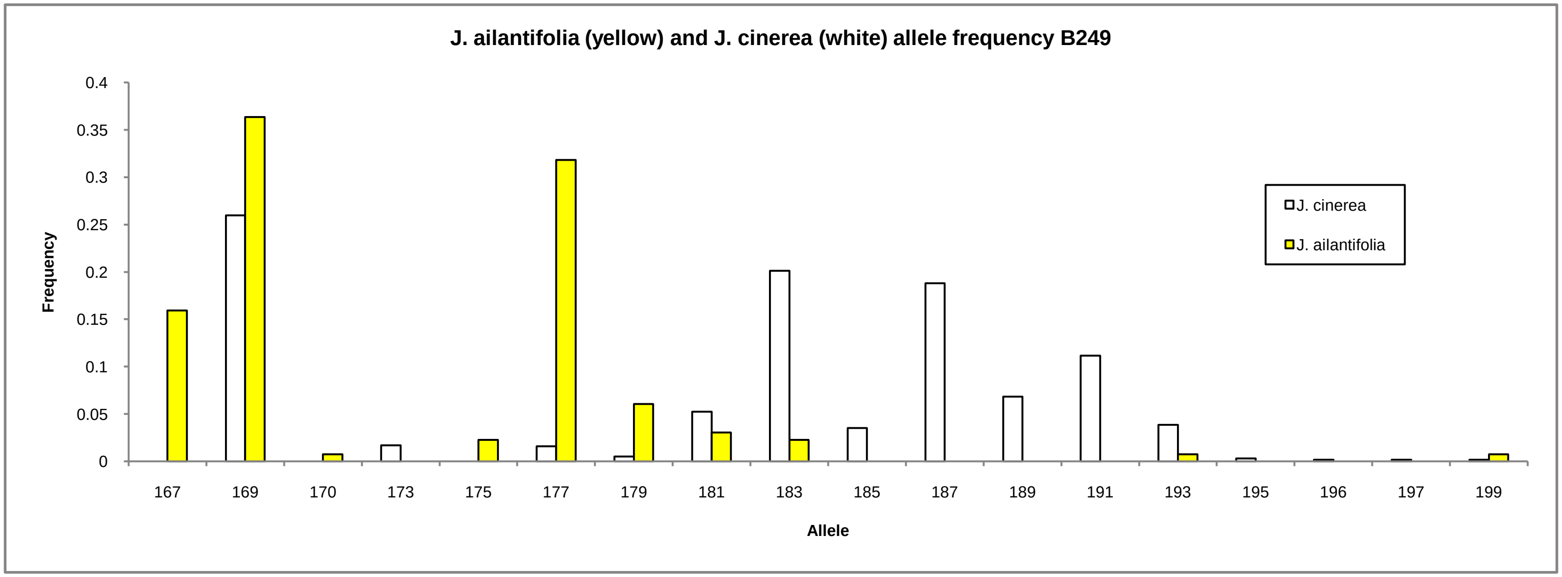
J. ailantifolia (yellow) and J. cinerea (white) allele frequency B249
Frequency
0.4
0.35
0.3
0.25
0.2
0.15
0.1
0.05
0
167
169
170
173
175
177
179
181
183
185
187
189
191
193
195
196
197
199
Allele
J. cinerea
J. ailantifolia

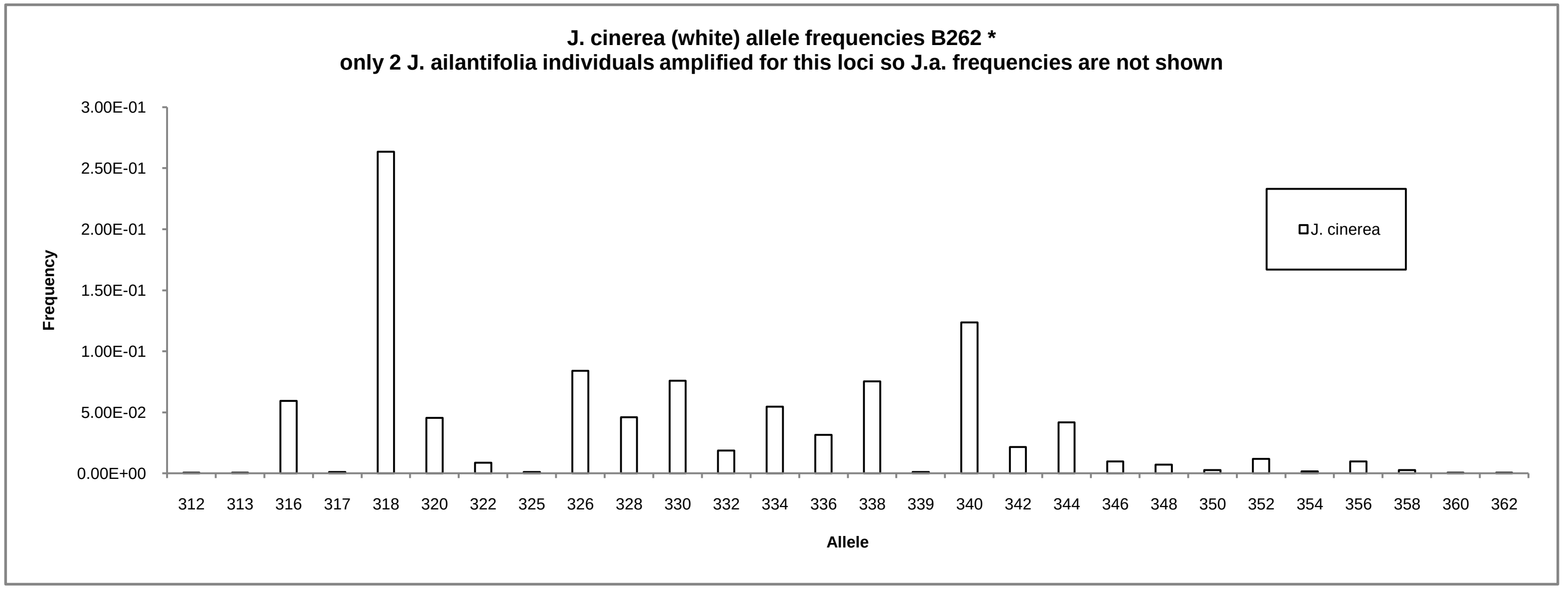
J. cinerea (white) allele frequencies B262 *
only 2 J. ailantifolia individuals amplified for this loci so J.a. frequencies are not shown
Frequency
3.00E-01
2.50E-01
2.00E-01
1.50E-01
1.00E-01
5.00E-02
0.00E+00
J. cinerea
312 313 316 317 318 320 322 325 326 328 330 332 334 336 338 339 340 342 344 346 348 350 352 354 356 358 360 362
Allele

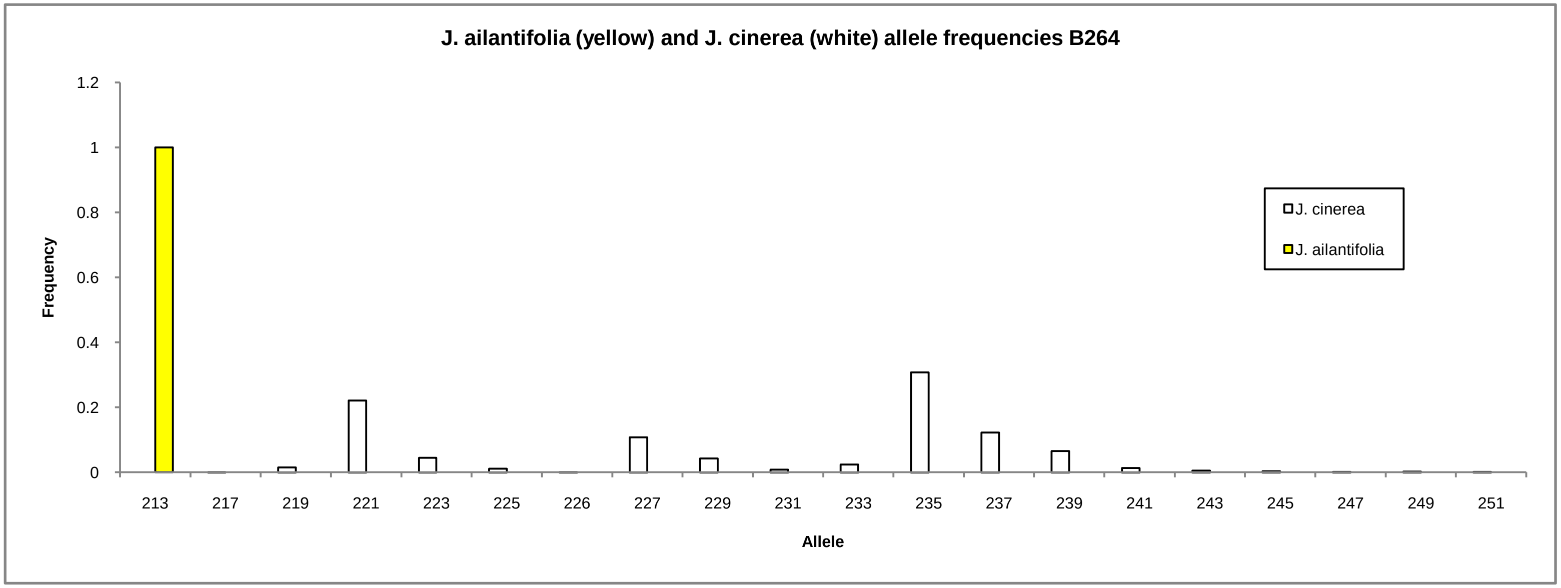
J. ailantifolia (yellow) and J. cinerea (white) allele frequencies B264
Frequency
1.2
1
0.8
0.6
0.4
0.2
0
J. cinerea
J. ailantifolia
213 217 219 221 223 225 226 227 229 231 233 235 237 239 241 243 245 247 249 251
Allele
