# Supplementary figures and images for: Human-impacted landscapes facilitate hybridization between a native and an introduced tree
Source: Evol Appl. 2012 Nov;5(7):720–31. doi: 10.1111/j.1752-4571.2012.00250.x (PMC3492897; doi:10.1111/j.1752-4571.2012.00250.x)

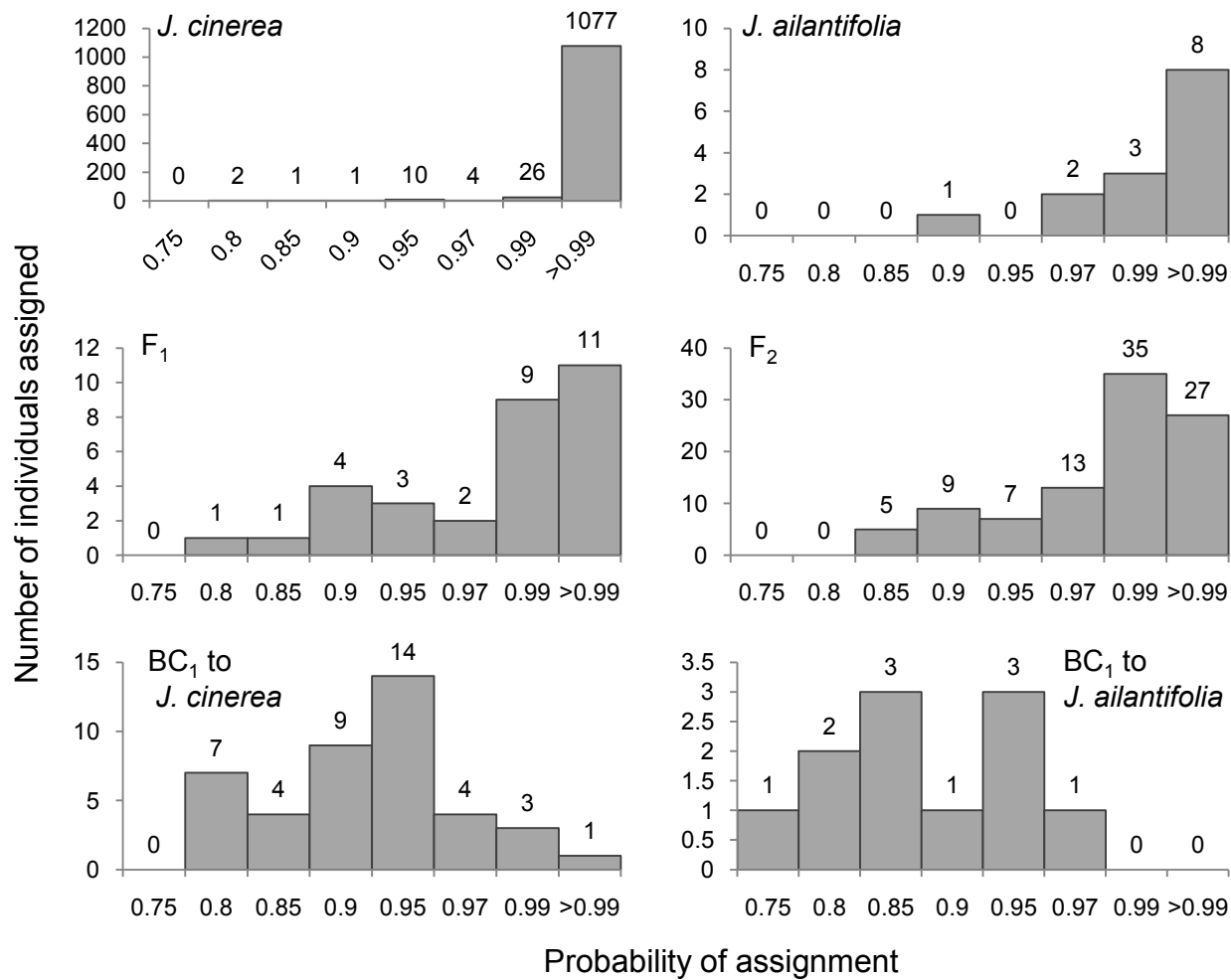

Supplement: Supplementary file 2 [file eva0005-0720-SD2.pdf]
