# Supplementary material for: Human-impacted landscapes facilitate hybridization between a native and an introduced tree
Source: Evol Appl. 2012 Nov;5(7):720–31. doi: 10.1111/j.1752-4571.2012.00250.x (PMC3492897; doi:10.1111/j.1752-4571.2012.00250.x)

Legend

- F1 blue
- F2 green
- BC_{JC} orange
- mix pink
- JA yellow
- JC white

*BC_{JA} not observed in these sites

Jericho Research Station, Vermont

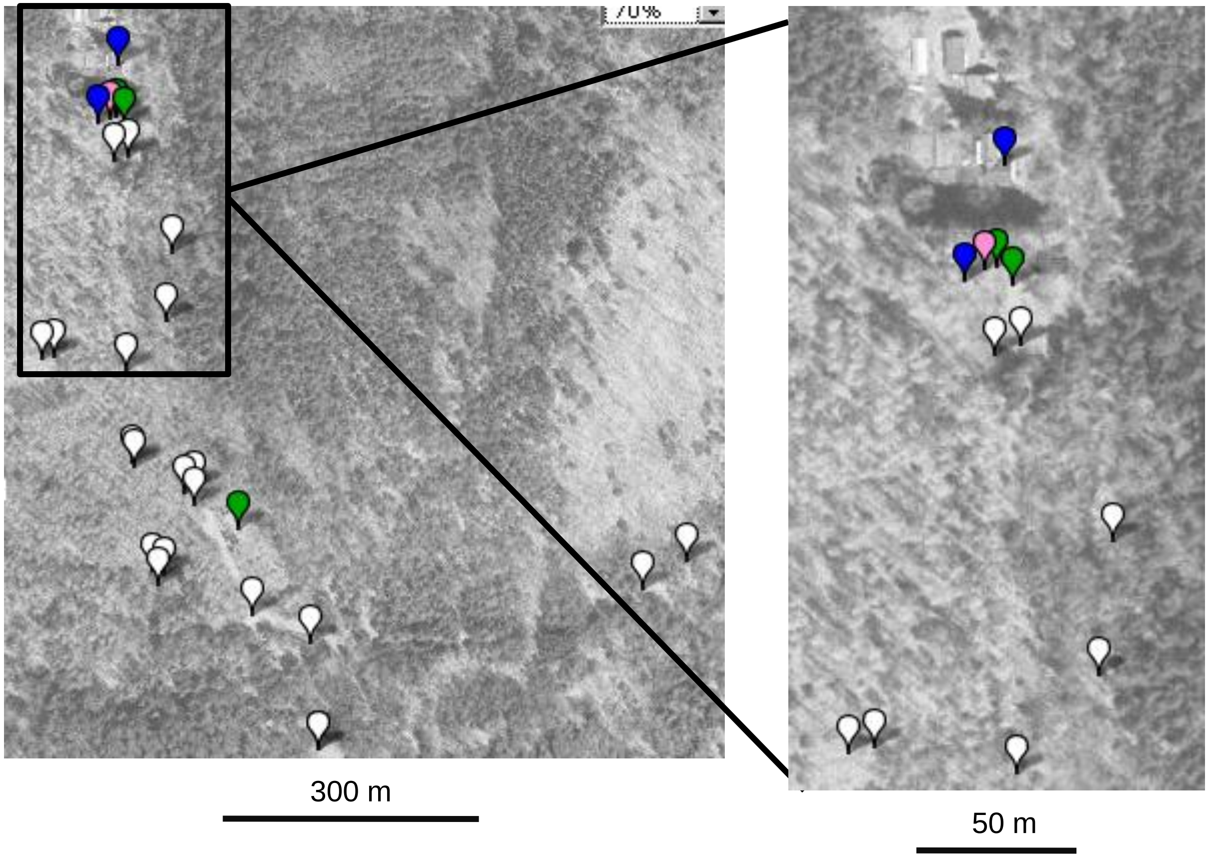

Allegheny National Forest, Pennsylvania

GR site

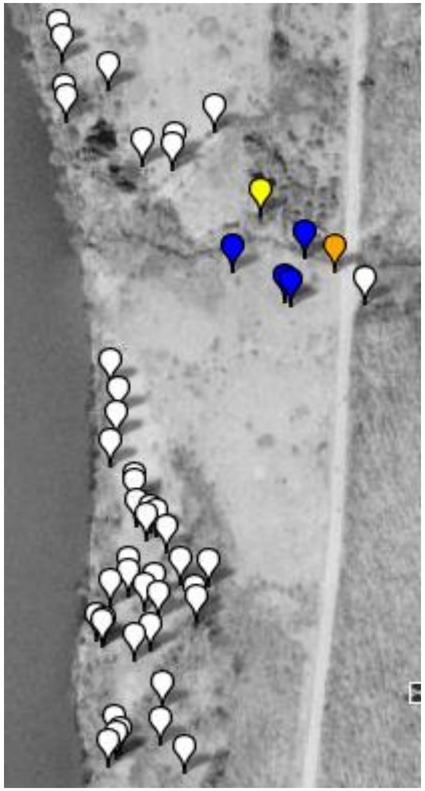

300 m

DD site

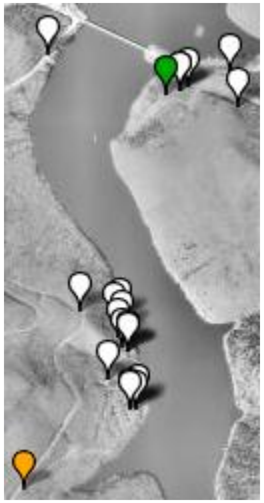

1 km

St Francis National Forest, Arkansas

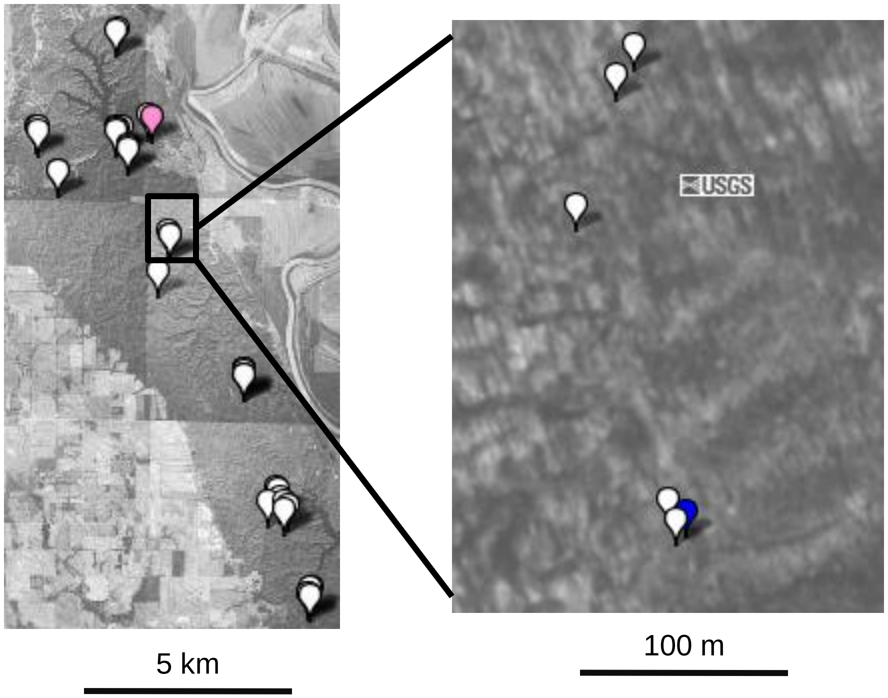

Mammoth Cave National Park, Kentucky

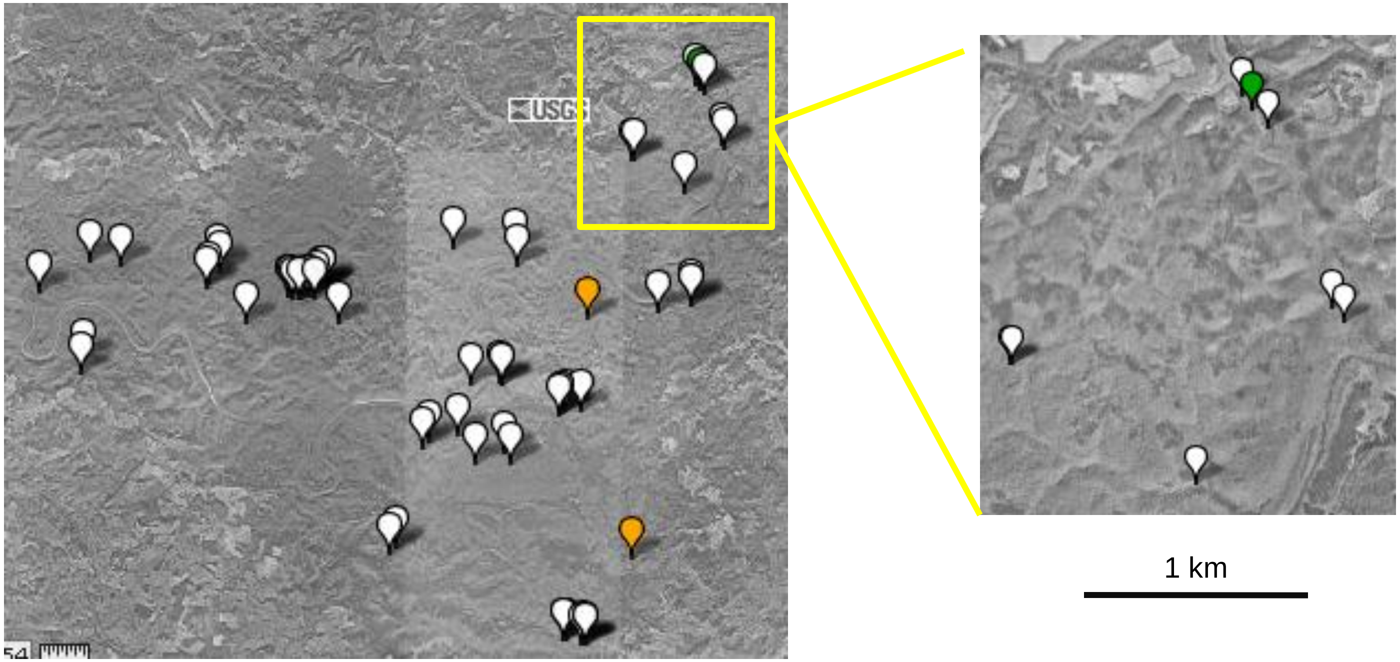

1 km

5 km

Butternut Valley, Tennessee

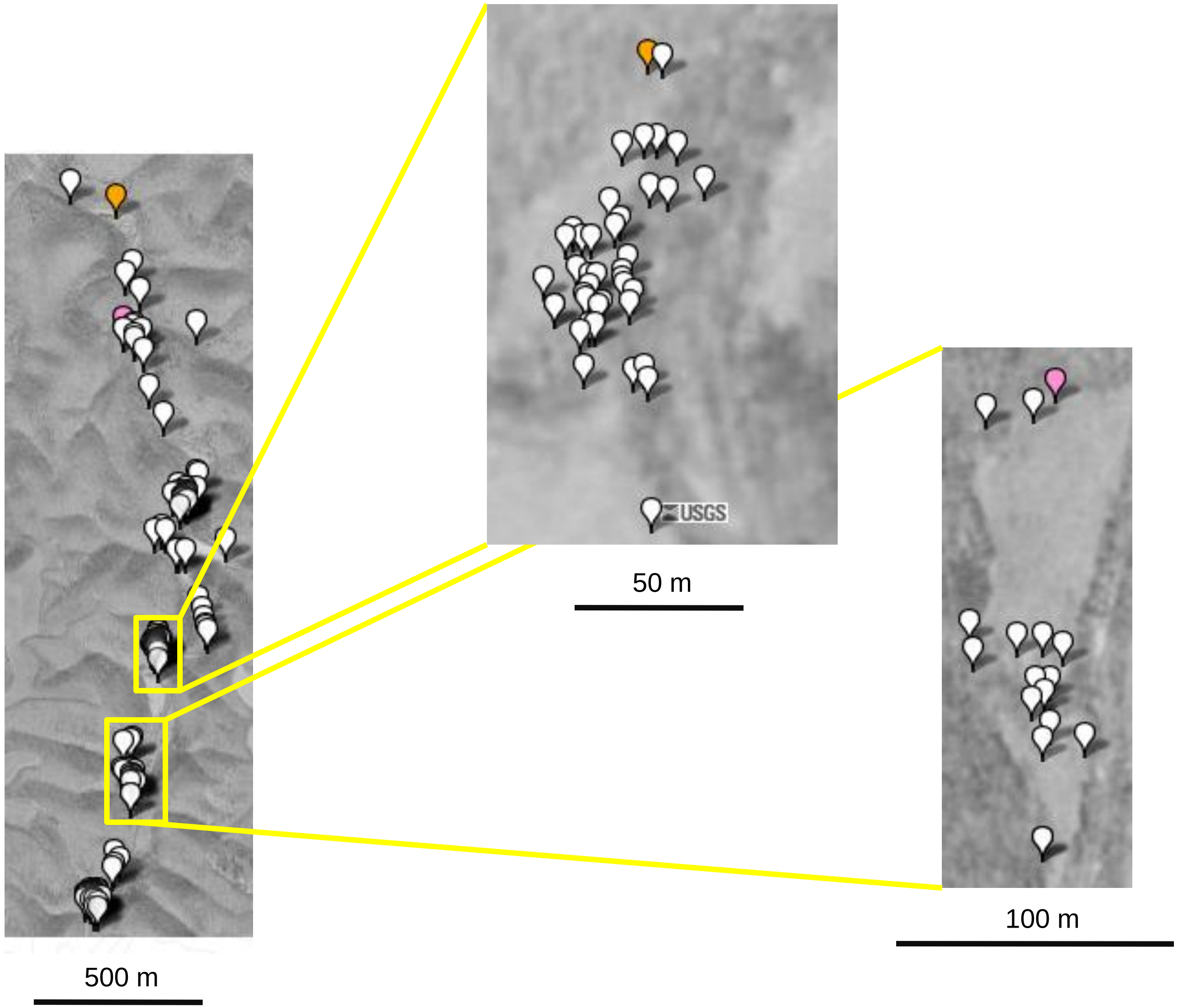

Supplement: Supplementary file 3 [file eva0005-0720-SD3.pdf]
