# Supplementary material for: Human-impacted landscapes facilitate hybridization between a native and an introduced tree
Source: Evol Appl. 2012 Nov;5(7):720–31. doi: 10.1111/j.1752-4571.2012.00250.x (PMC3492897; doi:10.1111/j.1752-4571.2012.00250.x)
Supplement: Supplementary file 4 [file eva0005-0720-SD4.pdf]

Supplemental Table 1

	prior	
	**uniform**	**jeffrey**
**JC**	1128	1121
**JA**	18	14
**F1**	107	96
**F2**	15	31
**BCJC**	25	42
**BCJA**	8	11
**mix**	62	48
**number hybrids**	217	228
