# Supplementary material for: Human-impacted landscapes facilitate hybridization between a native and an introduced tree
Source: Evol Appl. 2012 Nov;5(7):720–31. doi: 10.1111/j.1752-4571.2012.00250.x (PMC3492897; doi:10.1111/j.1752-4571.2012.00250.x)
Supplement: Supplementary file 5 [file eva0005-0720-SD5.pdf]

Supplemental Table 2. Note that while the 0.90 threshold shows slight advantage in accuracy, the 0.75 threshold shows a much greater advantage in effeciency and overall performance

**FOR 0.95 THRESHOLD**		**assigned**							
		**JC**	**JA**	**F1**	**F2**	**BCJC**	**BCJA**	**mix**	**TOTAL**
**simulated**	**JC**	**4957**	0	0	0	0	0	13	4970
	**JA**	0	**301**	0	0	0	0	29	330
	**F1**	0	0	**416**	0	0	0	84	500
	**F2**	0	0	1	**316**	4	6	173	500
	**BCJC**	6	0	0	0	**275**	0	219	500
	**BCJA**	0	2	1	0	0	**244**	53	300

TOTAL	4963	303	418	316	279	250	571
efficiency	0.99738	0.91212	0.832	0.632	0.55	0.81333	
accuracy	0.99879	0.9934	0.99522	1	0.98566	0.976	
performance	0.99618	0.9061	0.82802	0.632	0.54211	0.79381	

**FOR 0.90 THRESHOLD**		**assigned**							
		**JC**	**JA**	**F1**	**F2**	**BCJC**	**BCJA**	**mix**	**TOTAL**
**simulated**	**JC**	**4960**	0	0	0	0	0	10	4970
	**JA**	0	**314**	0	0	0	1	15	330
	**F1**	0	0	**457**	0	0	0	43	500
	**F2**	0	0	2	**344**	7	8	139	500
	**BCJC**	7	0	0	0	**367**	0	126	500
	**BCJA**	0	2	1	0	0	**353**	144	500

TOTAL	4967	316	460	344	374	362	477
efficiency	0.99799	0.95152	0.914	0.688	0.734	0.706	
accuracy	0.99859	0.99367	0.99348	1	0.98128	0.97514	
performance	0.99658	0.94549	0.90804	0.688	0.72026	0.68845	

**FOR 0.75 THRESHOLD**		**assigned**							
		**JC**	**JA**	**F1**	**F2**	**BCJC**	**BCJA**	**mix**	**TOTAL**
**simulated**	**JC**	**4968**	0	0	0	0	0	4	4972
	**JA**	0	**323**	0	0	0	4	3	330
	**F1**	0	0	**485**	0	0	1	14	500
	**F2**	0	0	3	**373**	20	35	69	500
	**BCJC**	8	0	1	2	**452**	0	37	500
	**BCJA**	0	2	7	0	0	**444**	47	500

TOTAL	4976	325	496	375	472	484	174
efficiency	0.9992	0.97879	0.97	0.746	0.904	0.888	
accuracy	0.99839	0.99385	0.97782	0.99467	0.95763	0.91736	
performance	0.99759	0.97276	0.94849	0.74202	0.86569	0.81461	
