# Supplementary material for: Human-impacted landscapes facilitate hybridization between a native and an introduced tree
Source: Evol Appl. 2012 Nov;5(7):720–31. doi: 10.1111/j.1752-4571.2012.00250.x (PMC3492897; doi:10.1111/j.1752-4571.2012.00250.x)
Supplement: Supplementary file 6 [file eva0005-0720-SD6.pdf]

Supplemental Table 3: Mean distance between *J. cinerea* individuals compared with distance between non *J.cinerea* individuals at six sites.

	Forested			Fragmented		
Mean distance (m)	SFNF	HS	MC	DD	JVT	GR
J. cinerea	7577	888	7922	1008	240	261
non- *J. cinerea*	8603	1372	8939	2976	142	56
P value, one-tailed	> 0.999[1]	0.913[2]	0.664[2]	> 0.999[1]	0.022[2]	< 0.001[2]

[1]t- test

[2]z-test
